# *P2RY14* Is a Potential Biomarker of Tumor Microenvironment Immunomodulation and Favorable Prognosis in Patients With Head and Neck Cancer

**DOI:** 10.3389/fgene.2021.670746

**Published:** 2021-07-08

**Authors:** Qingxiang Li, Le Xu, Yuke Li, Rong Yang, Qiao Qiao, Yifei Wang, Lin Wang, Yuxing Guo, Chuanbin Guo

**Affiliations:** Department of Oral and Maxillofacial Surgery, Peking University School and Hospital of Stomatology, National Center of Stomatology, National Clinical Research Center for Oral Diseases, National Engineering Laboratory for Digital and Material Technology of Stomatology, Beijing Key Laboratory of Digital Stomatology, Research Center of Engineering and Technology for Computerized Dentistry Ministry of Health, NMPA Key Laboratory Technology for Computerized Dentistry Ministry of Health, NMPA Key Laboratory for Dental Materials, Beijing, China

**Keywords:** *P2RY14*, head and neck cancer, tumor microenvironment, tumor-infiltrating immune cells, immunomodulation

## Abstract

The tumor microenvironment (TME) has a crucial role in tumor development, progression, and treatment response. Yet, the exact interaction between cancer biology and the TME is not fully understood. The following study analyzed the correlation between immune/stromal/estimate scores and survival prognosis in head and neck squamous cell carcinoma (HNSC) using a bioinformatic method. As a result, a predictive biomarker, UDP-glucose-specific G(i) protein-coupled P2Y receptor (P2RY14), was discovered. The potential role of P2RY14-driven signaling pathways in the immune-remodeling of TME was then investigated. Briefly, low immune scores were associated with unfavorable prognosis and clinical-stage, larger tumor size, and the down-regulation of *P2RY14* in HNSC patients. In addition, the survival analysis showed that HNSC patients with high expression had longer survival than patients with low expression from both TCGA databases and our own patients. We further discovered that *P2RY14* is involved in the immune activity in the TME of HNSC; a downregulation of *P2RY14* resulted in being an indicator for the conversion of TME status (from immune-dominant to metabolic-dominant status). The intersection analysis of genes co-expressed with *P2RY14* indicated that the T-cell receptor signaling pathway and PD-L1 expression and PD-1 checkpoint pathway were candidate signaling pathways driven by the *P2RY14* gene in HNSC. Further investigation of immune-associated signaling pathways regulated by *P2RY14* may help HNSC patients gain higher immunotherapy benefits.

## Introduction

Head and neck squamous cell carcinoma (HNSC) is one of the most common malignancies of the head and neck region and a sixth leading cancer worldwide (Johnson et al., 2020). More than 400,000 people die from HNSC every year (Chen et al., 2016; Bray et al., 2018). Although great advancements have been made in the management of HNSC, the overall 5-year survival rate of HNSC patients remains poor. Neck lymph node metastasis, local recurrence, or resistance to radiation and chemotherapy often occur in patients with HNSC (Pisani et al., 2020). A combination of chemotherapy, targeted therapy, radiation therapy, and immunotherapy are mandatory for HNSC patients with advanced-stage disease (Manukian et al., 2019). For example, programmed cell death protein 1 (PD1) inhibitors combined with chemotherapy has been recently approved by the FDA for recurring or metastatic HNSC. However, the response rate of PD1 immunotherapy in the clinical treatment of head and neck cancer still remains unsatisfactory, and only a small proportion of patients experience durable benefit (Jia et al., 2020; Johnson et al., 2020). Hence, it is essential to explore new molecular markers that have a prognostic value in influencing the immune response of HNSC patients.

The tumor microenvironment (TME) has a crucial role in tumor development, progression, and treatment response. This environment includes the surrounding blood vessels, immune cells, fibroblasts, signaling molecules, and the extracellular matrix (Whiteside, 2008; New et al., 2017). Studies have suggested that TME cells can affect the epithelial-mesenchymal transition (EMT) process of cancers through autocrine or paracrine ways (Fiori et al., 2019; Goulet et al., 2019). For example, cancer-associated fibroblasts can secrete IL-6 to promote tumor EMT, and at the same time, promote aggressive phenotypes of bladder cancer and breast cancer cells (Su et al., 2018; Goulet et al., 2019). Stromal cells and immune cells have an important role in the diagnosis and prognosis of tumors (Jia et al., 2018). Therefore, understanding the molecular function and composition of TME is essential for the effective management of cancer progression.

UDP-glucose-specific G(i) protein-coupled P2Y receptor (*P2RY14*) is a protein-coding gene that has an important role in signaling by G-protein-coupled receptors (GPCR) and peptide ligand-binding receptors. In stem/progenitor cells, P2RY14 inhibits cell senescence by monitoring and responding to the extracellular manifestations of tissue stress (Cho et al., 2014). A selective high-affinity antagonist of the P2RY14 has been reported to inhibit UDP-glucose–stimulated chemotaxis of human neutrophils (Barrett et al., 2013). Another study reported that P2RY14 inhibition protects mice against ischemic acute kidney injury by reducing neutrophil and monocyte renal infiltration (Battistone et al., 2020). So far, few studies have reported on the role of P2RY14 in tumors.

Over the last two decades, several genome-wide gene expression repertoires, such as The Cancer Genome Atlas (TCGA), have been established to explore the impact of tumor genetic composition on clinical outcomes (Zhou et al., 2020). In this study, the TME-related genes were obtained from the HNSC datasets in the TCGA database. Consequently, the ESTIMATE algorithm and CIBERSORT computational method were used to analyze the corresponding immune/stromal/estimate scores. As a result, a predictive biomarker P2RY14 was identified. *P2RY14* has shown to be correlated with the survival, classification of TNM stages, and tumor-infiltrating immune cells (TICs) in HNSC patients. Finally, Gene Set Enrichment Analysis (GSEA) was used to explore the molecular mechanism of *P2RY14* in the immune modulation of head and neck cancer.

## Materials and Methods

### Raw Data

RNA-seq data of 543 HNSC cases (normal sample, 44 cases; tumor sample, 499 cases) and corresponding clinical data (age, gender, TNM staging, survival-time, and status) were downloaded from the cBioPortal^1^. The samples’ stromal/immune/estimate scores were calculated using the ESTIMATE algorithm^2^.

### Survival Analysis

Receiver operating characteristic (ROC) curve analysis was used to determine the cut-off score of ImmuneScore and StromalScore and the cut-off expression level of *P2RY14*. The survival curve was drawn using the “survminer” and “survival” R packages. This relationship was verified using a log-rank test. The *p*-value < 0.05 was considered to be statistically significant (Jia et al., 2018). These analyses illustrated the relationship between stromal/immune/estimate scores or *P2RY14* expression level and overall patient survival.

### Differential Expression Analysis

Four hundred ninety-nine tumor samples were classified into a high score or low score group, depending on the comparison to the cut-off score regarding ImmuneScore and StromalScore. Differential expression analysis was performed on the count matrix of the sample using the “limma” R-package. The screening conditions for the differential genes were performed as follows: |log_2_(fold change)| > 4, false discovery rate (FDR) < 0.05. Heat maps of differential genes were drawn using the “pheatmap” R-package.

### Enrichment Analysis and Protein-Protein Interaction Network

R language was used to perform functional enrichment analysis of the differentially expressed genes (DEGs), including Kyoto Encyclopedia of Genes and Genomes (KEGG) and Gene Ontology (GO). PPI network was constructed using the Cytoscape software. The top 30 genes ordered according to the number of nodes (from large to small) are shown in the plot (Zhou et al., 2020).

### COX Regression Analysis

R-language loaded with package survival was used for univariate COX regression. The top 30 genes ordered by *p*-value from small to large in univariate COX are shown in the plot.

### Patients

Oral squamous cell carcinoma (OSCC) patients who underwent surgical resection at the Peking University School and Hospital of Stomatology during 2014–2016 were enrolled in the current study. Patients with diabetes, hyperthyroidism, and other metabolic diseases were excluded. Finally, 94 patients [51 males and 43 females, mean age of 59 years (age range: 27–88)] were included in the study. Telephone follow-up was performed to analyze the survival trends among these patients.

The Ethics Committee of Peking University School and Hospital of Stomatology (NO. PKUSSIRB-201839134) approved this study. Informed consent was obtained from all patients.

### Immunohistochemistry

The tissue sections of 94 OSCC patients were collected, deparaffined, and rehydrated in xylene and a gradient ethyl alcohol series. Antigen retrieval was performed in 0.01 M sodium citrate buffer (pH = 6.0) and the samples were treated with 3% (w/v) hydrogen peroxide for 10 min. Anti-P2RY14 antibody (1:1,000, ab140896, Abcam, United States) and horseradish peroxidase-labeled secondary antibody (1:100, ZSbio, China) were then used for staining. All images were analyzed with an optical microscope (Olympus). Five representative fields separated over the tumor tissue were randomly selected for statistical analysis.

### Tumor-Infiltrating Immune Cells Profile

CIBERSORT computational method was used for the estimation of the TIC abundance profile. R language was used to perform the correlation analysis between the mRNA expression data of *P2RY14* in TCGA HNSC tumor samples and nine kinds of TICs.

### Gene Set Enrichment Analysis

Kyoto Encyclopedia of Genes and Genomes pathway gene set (c2.cp.kegg.v7.2.symbols.gmt) was used as the target sets. The software gsea-4.1.0 was downloaded from Broad Institute to perform the analysis. The whole transcriptome of all tumor samples was used for GSEA. Only the gene sets with NOM *p* < 0.05, FDR *q* < 0.25 was considered as significant (Su et al., 2018).

### Statistical Analysis

The Kaplan–Meier method was used for survival analysis. One-way ANOVA was used to compare the *P2RY14* expression level among the different clinical/T/N stages from all patients. A *p*-value < 0.05 was considered to be statistically significant.

## Results

### Immune Scores Associated With Survival Prognosis *via* the Tumor Size in the TNM Staging System

**FIGURE 1:**
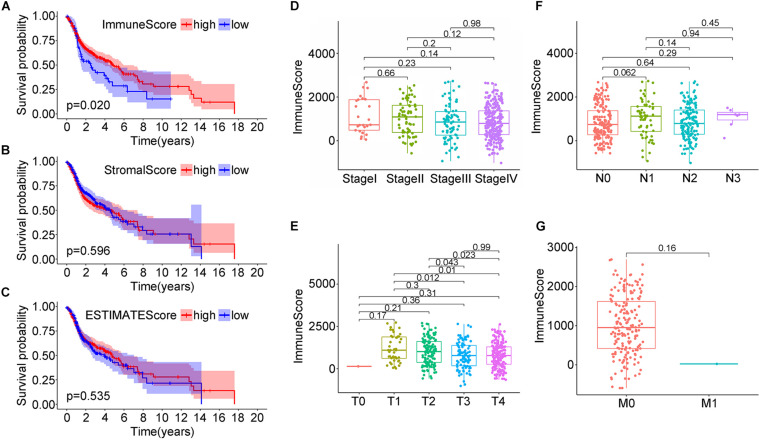
Correlation analysis of head and neck squamous cell carcinoma (HNSC) patients survival and stromal score/immune score/estimate score distribution from the TCGA database. **(A–C)** Kaplan–Meier survival analysis for HNSC patients grouped by immune score, stromal score, and estimate score. **(D–G)** Distribution of immune score in clinical stage, T classification, N classification, and M classification.

The RNA-seq gene expression matrix and clinical information data of 499 HNSC patients were downloaded from cBioPortal. 26.65% were male, and 73.35% were female (Supplementary Table 1). The stromal, immune, and estimate scores of all samples were obtained using the ESTIMATE algorithm; the score ranges were −1,974.56 to 1,959.79, −1,016.77 to 2,809.25, and −2,861.51 to 4,654.11, respectively. To analyze the potential relationship between the scores and the overall survival, patients were classified into high-score and low-score groups. For the immune scores, the Kaplan–Meier survival curve showed a higher survival rate in the high score group than the low score group (Figure 1A, *p* = 0.020 in the log-rank test); while no difference was observed between stroma and estimate score groups (low vs. high) (Figures 1B,C).

Next, the correlation between the immune scores and the clinical factors of HNSC patients was analyzed to explore how immune scores affect HNSC patients’ prognosis. When considering clinical staging (T), the median immune scores in stage I were the lowest while they were the highest in stage II; the order of immune scores was: StageII > StageIII > StageIV > StageI, but the difference was not statistically significant (Figure 1D, *P* > 0.05). When considering the tumor size, the score decreased with the tumor size, and the relationships between the median immune scores of T1 and T3, T1 and T4, T2 and T3, T2 and T4 were statistically significant (*P* = 0.012; *P* = 0.01; *P* = 0.043; *P* = 0.023, respectively; Figure 1E). When considering lymph node staging (N), the relationships between the median immune scores of the four stages were nearly similar (*P* > 0.05), namely: N1 > N3 > N2 > N0 (Figure 1F). Finally, when considering distant metastasis (M), the immune median scores were: M0 > M1, without statistical significance (Figure 1G, *P* > 0.05). This data suggested a strong correlation between immune scores and the tumor sizes in the TNM staging system of the HNSC patients.

### Differential Expression and Enrichment Analysis of HNSC Cases Based on Stromal and Immune Scores

**FIGURE 2:**
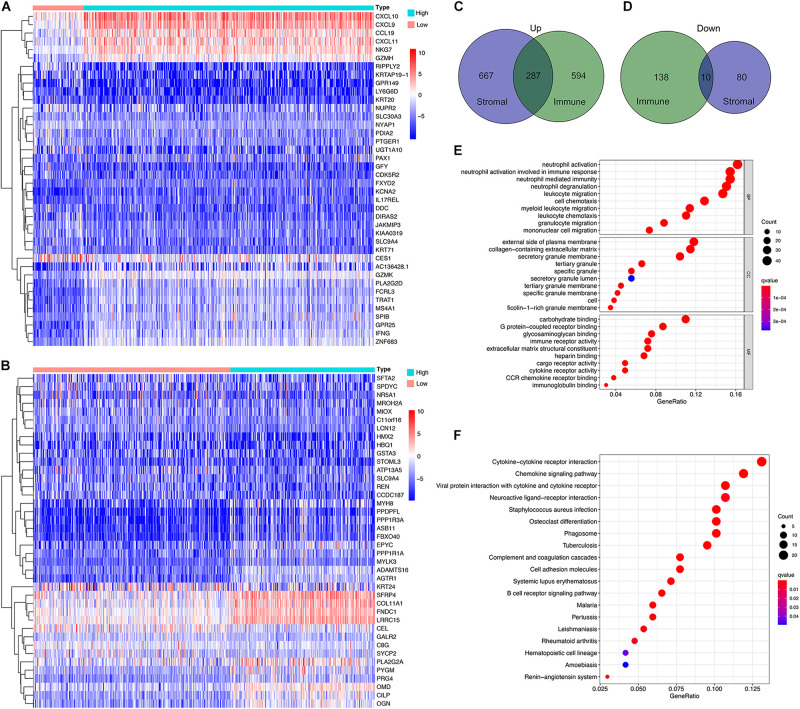
Profile of differential expressed genes (DEG)s and functional enrichment analysis based on the stromal and immune scores. **(A,B)** Heatmap for DEGs generated by comparing the high score with low score group for Immune Scores and Stromal Scores. The row name of the heatmaps is the gene name. **(C,D)** Common up-regulated, and down-regulated DEGs shared by Immune Score and Stromal Score in the Venn plots. **(E,F)** GO and KEGG enrichment analysis for DEGs.

The DEG analysis of all RNAseq data (499 HNSC cases in TCGA database) was performed to examine the relationship between the stromal and/or immune scores and the gene expression profile of the samples. The heat map of the stromal/immune scores (low vs. high group) revealed differential gene expression profiles between the samples: stromal scores: 954 genes were up-regulated, and 90 were down-regulated; immune scores: 881 were up-regulated and 148 genes were down-regulated (Figures 2A,B). As the Venn diagram shows, there are 287 identical up-regulated genes and 10 identical down-regulated genes (Figures 2C,D and Supplementary Tables 2–4).

Next, functional enrichment analysis, including BP, CC, MF using GO, and the analysis of the signaling pathways using KEGG, were performed. GO functions were mainly enriched in extracellular matrices, and carbohydrate or GPCR binding, and inflammatory and immune responses (Figure 2E), while KEGG pathways were mainly enriched in cytokine-cytokine receptor interaction and chemokine signaling pathway (Figure 2F).

### Hub Genes of DEGs With Prognostic Values in HNSC Cases

**FIGURE 3:**
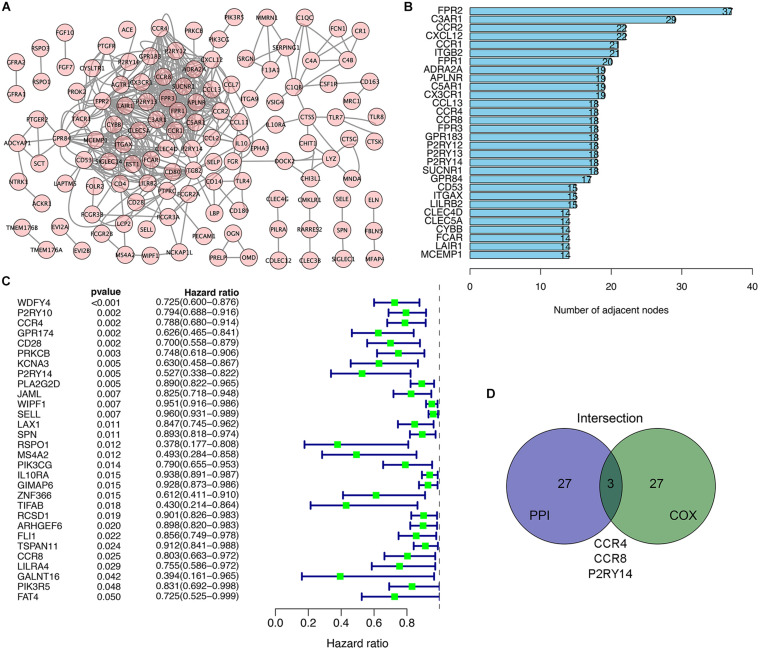
Intersection analysis of top genes that derived protein-protein interaction (PPI) network and univariate COX processing. **(A)** PPI network constructed with the nodes with interaction confidence value > 0.90. **(B)** The top 30 genes ordered according to the number of adjacent nodes (from large to small). **(C)** Univariate COX regression analysis with DEGs. Listing the top 30 significant factors with *p* < 0.05 (from small to large). **(D)** The common factors shared by the top 30 genes in PPI and top 30 significant factors in univariate COX.

We used the STRING network tool to analyze the interrelationship between genes with prognostic value to construct a PPI network of genes with prognostic values (Figure 3A). The number of adjacent nodes of the top 30 genes most closely related to prognosis values was between 37 and 14 (Figure 3B). Univariate Cox regression analysis for HNSC patients’ survival was performed to determine the prognosis-associated factors among 297 DEGs (Figure 3C). Then, the intersection analysis between the top 30 genes from leading nodes in the PPI network and the top 30 factors ranked by the *p*-value of univariate COX regression was carried out. Only three factors (*CCR4*, *CCR8*, and *P2RY14*) were overlapping from the above analyses (Figure 3D).

In a recent biometric analysis study, the TME-related genes *CCR4*, *CCR8*, and UDP-glucose-specific G(i) protein-coupled P2Y receptor (*P2RY14*) were suggested as prognostic factors in HNSC datasets (Meng et al., 2021). However, no difference in the expression of CCR4 was found between normal tissues and tumor tissues. The expression of CCR8 was lower in normal tissues than in tumor tissues; but the survival analysis suggested CCR8 is positively correlated with prognosis (the higher the expression of CCR8, the better the prognosis). The above information indicates that the role of *CCR4* and *CCR8* in the microenvironment of head and neck cancer is not very clear. On the other hand, this data suggests that *P2RY14* play an important role in the regulation of the tumor immune microenvironment.

### The Correlation of *P2RY14* Expression With the Survival and Classification of TNM Stages in HNSC Patients

**FIGURE 4:**
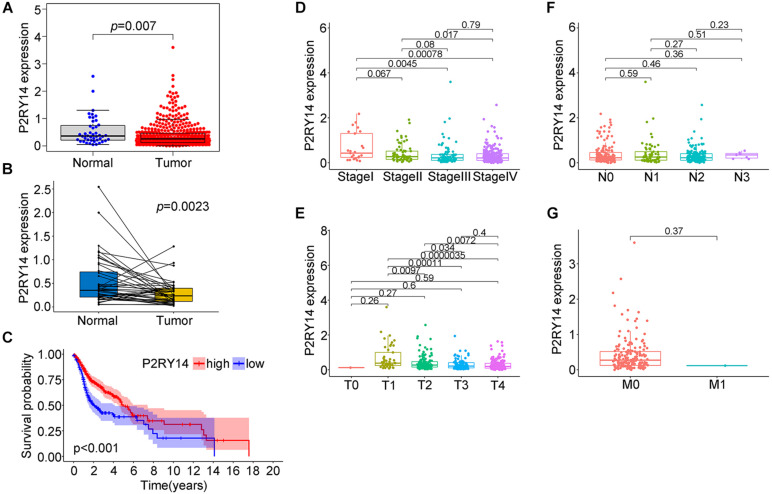
Expression of P2RY14 in samples of HNSC patients from the TCGA database and its correlation with survival and TNM staging distribution. **(A)** Differentiated expression of P2RY14 in normal and tumor samples. **(B)** Paired differentiation analysis of P2RY14 in the normal and tumor sample derived from the same patient. **(C)** Survival analysis for HNSC patients with different P2RY14 expression. **(D–G)** Distribution of P2RY14 expression level in clinical stage, T classification, N classification, and M classification.

*P2RY14* is highly expressed in the intestine, adipose tissue, stomach, and placenta (Jokela et al., 2014; Shah et al., 2018). In the bone marrow hematopoietic stem cells (HSCs) subpopulations, P2RY14 promotes regenerative responses after injury. Moreover, increased senescence of HSCs in response to aging, chemotherapy, radiation, and other environmental stresses have been found in *P2RY14* knockout mice (Lazarowski and Harden, 2015). In the present study, we extracted the data from the TCGA data in order to explore the expression level of *P2RY14* in normal healthy tissue and HNSC tissues. Wilcoxon rank-sum test revealed a significantly lower expression of *P2RY14* in the tumor samples compared to normal samples (Figure 4A). Similar results were found in the pairing analysis between the tumor tissues and normal healthy tissue derived from the same patients (Figure 4B).

In this study, HNSC samples were grouped into *P2RY14* high-expression group and *P2RY14* low-expression group. The survival analysis showed that HNSC patients with high expression had longer survival than patients with low expression from TCGA databases (Figure 4C), thus suggesting that *P2RY14* were positively correlated with the prognosis of HNSC. Then, the analysis of *P2RY14* combined with clinical characteristics was performed. In particular, the expression of *P2RY14* was reduced along with the progression of clinical stages and tumor size (Figures 4D–G).

**FIGURE 5:**
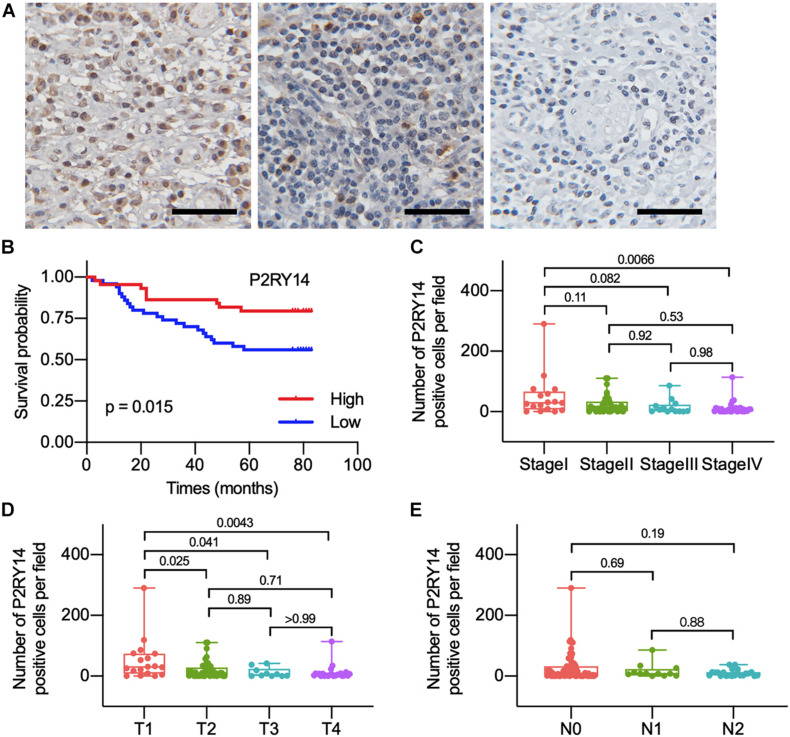
The differentiated expression of P2RY14 in samples and correlation with survival and distribution of OSCC patients. **(A)** Representative immunohistochemical staining images of P2RY14 in tumor stroma from HNSC patients (scale bar: 50 μm). **(B)** Survival analysis for HNSC patients with different P2RY14 expression. **(C–E)** Distribution of P2RY14 expression level in clinical stage, T classification, and N classification.

To confirm the association between the expression of P2RY14 in tumor tissues and the clinicopathological characteristics of HNSC. The tumor specimens and corresponding clinicopathological information were obtained from 94 OSCC patients. Immunohistochemical staining results suggested that P2RY14 was mainly expressed in tumor stroma tissue (Figure 5A and Supplementary Figure 1). The survival analysis showed that OSCC patients with high-expression of P2RY14 had a more favorable prognosis than patients with low expression of P2RY14 (Figure 5B). In particular, the reduction of P2RY14 expressions along with the progression of clinical stages and tumor size in the TNM staging was consistent with the conclusion from the above database analysis (Figures 5C–E).

### Correlation of *P2RY14* Expression With the Proportion of TICs

**FIGURE 6:**
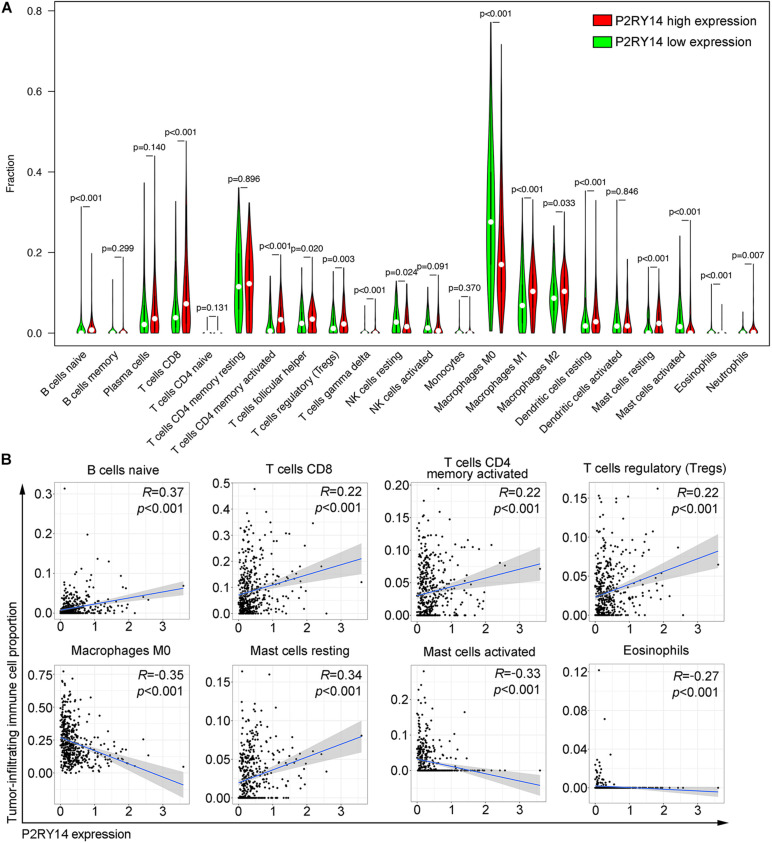
Correlation of tumor-infiltrating immune cells proportion with P2RY14 expression. **(A)** Violin plots showing the ratio differentiation of 22 kinds of immune cells between HNSC tumor samples with high or low P2RY14 expression. **(B)** Scatter plot showing the correlation of eight kinds of tumor-infiltrating immune cells proportion with the P2RY14 expression.

CIBERSORT algorithm was applied to analyze the proportion of tumor-infiltrating immune subsets and to further confirm the correlation of *P2RY14* expression with the immune microenvironment. Twenty-two immune cell profiles in HNSC samples were constructed. The results showed that 15 TICs were correlated with the expression of *P2RY14* (Figure 6A). Among them, five TICs were positively correlated with *P2RY14* expression, including naïve B cells, CD8 + T cells, activated memory CD4 + T cells, Tregs, and resting mast cells; three TICs were negatively correlated with *P2RY14* expression, including M0 macrophage, activated mast cells and eosinophils (Figure 6B). These results further suggested that *P2RY14* affects the immune activity of TME. *P2RY14* has been reported to inhibit UDP-glucose-stimulated chemotaxis of human neutrophils, which prompted further analysis of the relationship between *P2RY14* gene expression and neutrophil levels in head and neck cancer (Scrivens and Dickenson, 2006). In this study, we found a significant correlation between the two after analyzing three different algorithms (Supplementary Figure 2).

### *P2RY14* Has the Potential to Be an Indicator of TME Modulation

**FIGURE 7:**
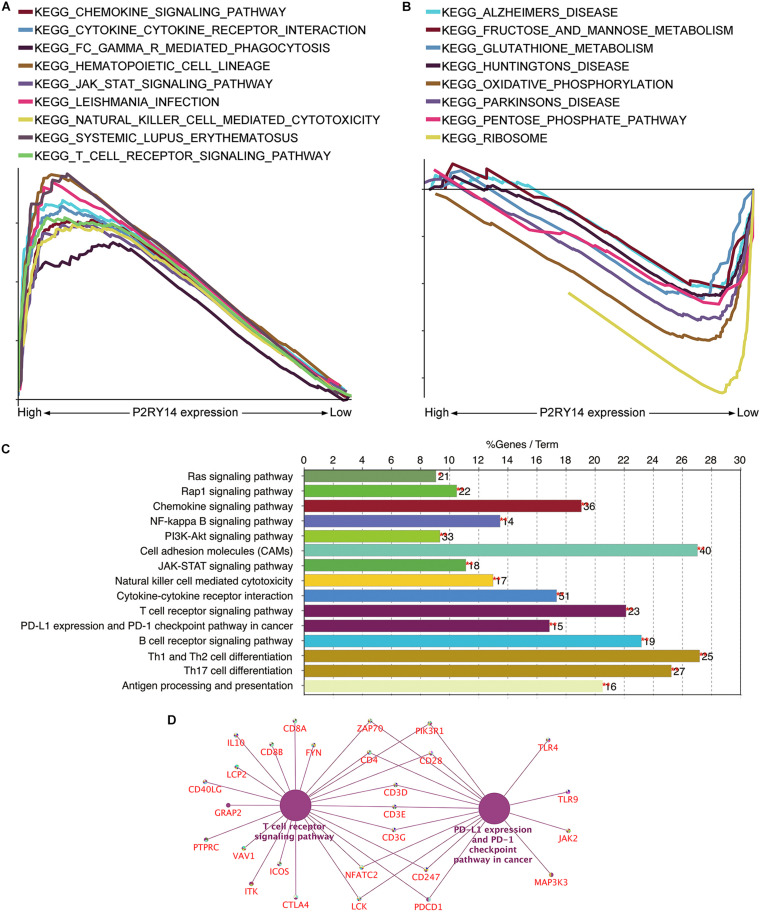
Analyses of the functional role of P2RY14 gene in the tumor microenvironment (TME) and the underlying regulatory genes in HNSC. **(A,B)** The enriched gene sets in HALLMARK by the samples with high or low P2RY14 expression. **(C)** KEGG pathway analysis of the genes co-expressed with P2RY14. **(D)** The genes co-expressed with P2RY14 in the T-cell receptor signaling pathway and PD-L1 expression and PD-1 checkpoint pathway in cancer.

Given that levels of *P2RY14* were negatively correlated with the survival and TNM stages of HNSC patients, GSEA was applied to the high-expression and low-expression groups compared with the median level of *P2RY14* expression, respectively. As shown in Figure 7A, the genes in the *P2RY14* high-expression group were mainly enriched in immune activities such as natural-killer-cell-mediated cytotoxicity and T-cell-Reporter signaling pathway. Metabolic pathways, including fructose and mannose metabolism, glutathione metabolism, oxidative phosphorylation, pentose phosphate pathway, and ribosome pathways, were mainly enriched in the low-expression group (Figure 7B).

Kyoto Encyclopedia of Genes and Genomes pathway analysis of the genes co-expressed with *P2RY14* was performed to see whether P2RY14 had a role in immune-related activities. Results showed the genes were enriched in natural-killer-cell-mediated cytotoxicity, T cell receptor signaling pathway, and PD-1 expression, and PD-1checkpoint pathway in cancer (Figure 7C).

Finally, the intersection analysis of genes co-expressed with *P2RY14* in T cell receptor signaling pathway and PD-L1 expression and PD-1 checkpoint pathway in cancer was carried out, and the candidate genes driven by the *P2RY14* gene in HNSC were enriched in *Zap70, PIK3R1, CD4, CD28, CD3D/E/G, CD247, NFATC2, LCK*, and *PDCD1* (Figure 7D).

## Discussion

In this paper, the ESTIMATE algorithm was used to calculate the stromal/immune scores based on RNA-seq data of HNSC obtained from the TCGA database. The HNSC samples with high immune scores showed a favorable prognosis compared with the low immune score group. Through differential and downstream analysis, 287 up-regulated genes and 10 down-regulated genes were obtained. *P2RY14* has been identified as a key gene involved in the overall survival and the immune activity in the TME of HNSC.

The TME can affect the behavior of cancerous cells and their response to treatment (Wang et al., 2018). Pancreatic cancer cells can produce signaling molecules to recruit or activate stromal and immunosuppressive cells, such as Tregs, MDSCs, TAMs, and PSCs, even at an early stage. The TME established by this procedure has a vital role in evolutionary and ecological processes in pancreatic cancer (Ren et al., 2018). The correlation between interleukin (IL)-6/IL-8-mediated M1 macrophage activity and CD10 expression could potentially explain the poor prognosis of HNSC (Li et al., 2020). Therefore, the exploration of intratumor immunity and stromal scores is beneficial to provide valuable promising guidance for tumor immunotherapy.

Immune checkpoint inhibitors, pembrolizumab, and nivolumab, which can hinder the inhibitory interaction between programmed cell death protein 1 (PD-1) and its ligand PD-L1, have been explored in patients with advanced/metastatic HNSC (Forster and Devlin, 2018). It was reported that the response rate of immunotherapy treatment in patients with advanced HNSC was only 18% at 9 months, and the benefits of responses were fairly limited (Chow et al., 2016). Therefore, obtaining the signaling information involved in the activation of cytotoxic T lymphocytes (CTL) in the microenvironment of head and neck cancer helps to improve the understanding of the interaction between cancer and the host immunity. It may be possible to develop potential biomarkers for selecting immunotherapy responding patients and assist in monitoring immunotherapy response. This study analyzed the TME-related genes in HNSC through the functional enrichment analysis of HNSC samples in the TCGA database using an ESTIMATE algorithm. The decreased expression of *P2RY14* was significantly associated with advanced clinicopathological characteristics (clinical stage and tumor size) and poor prognosis, thus suggesting that *P2RY14* might be a potential prognostic marker for HNSC.

A recent article used TCGA data analysis and found that *P2RY14* expression is down-regulated in lung cancer tissues (Wang et al., 2020). In this study, we further analyzed the possible role of *P2RY14* in the immune status of head and neck cancer TME. During the analysis of tumor-infiltrating immune subsets using the CIBERSORT algorithm, eight TICs were positively or negatively correlated with *P2RY14* expression. The samples with high *P2RY14* expression were mainly enriched in immune activities, while the samples with low *P2RY14* expression were mainly involved in metabolism activities. These results implied that P2RY14 might participate in the status conversion of TME from immune-dominant to metabolic-dominant. Yet, the exact mechanisms through which P2RY14 participates in the immunomodulation of HNSC remain unclear. P2RY14 could trigger the innate mucosal immunity of the female reproductive tract *via* the induction of IL-8 (Arase et al., 2009). By KEGG analysis of genes co-expressed with *P2RY14*, we found that the T-cell receptor signaling pathway and PD-L1 expression, and PD-1 checkpoint pathway are potential signaling candidates driven by P2RY14.

To sum up, our data showed that the balance between vigorous glycolysis metabolism and typical tumor pathways affects the immunity status. The disorder in the balance could be reflected by the correlation of low *P2RY14* expression with metabolism. Yet, the regulatory relationship between *P2RY14* and TIC in HNSC needs to be further explored. Accordingly, the downregulation of *P2RY14* along with the advancing stage of HNSC, the transformation of TME from predominant to dominant status, and the reduction of antitumor TICs supported that P2RY14 might have an antitumor role in HNSC. Therefore, further investigations, including the analysis of *P2RY14* expression and the activity level of T-cell signaling and PD-1 signaling in HNSC, are warranted.

## Data Availability Statement

The original contributions presented in the study are included in the article/Supplementary Material, further inquiries can be directed to the corresponding author/s.

## Ethics Statement

The studies involving human participants were reviewed and approved by the Ethics Committee of Peking University School and Hospital of Stomatology (No. PKUSSIRB-201839134). The patients/participants provided their written informed consent to participate in this study.

## Author Contributions

QL, LX, YG, and CG wrote, conceived, and designed the experiments. RY and QQ collected the samples. QL, LX, YL, and YG analyzed and interpreted the data. All authors read and approved the final manuscript.

## Conflict of Interest

The authors declare that the research was conducted in the absence of any commercial or financial relationships that could be construed as a potential conflict of interest.

## Footnotes

1 http://www.cbioportal.org/

2 https://r-forge.r-project.org

## Supplementary Material

Supplementary Figure 1 Representative immunohistochemical staining images of P2RY14 in tumor cells from HNSC patients (scale bar: 50 μm).

Supplementary Figure 2 Scatter plots showing the correlation of P2RY14 and neutrophils in head and neck cancer in the database using TIMER, MCPCOUNTER, and QUANTISEQ methods.

Supplementary Table 1 Differential gene expression based on stromal scores.

Supplementary Table 2 Differential gene expression based on immune scores.

Supplementary Table 3 Identical up and down-regulate differential genes from stromal and immune groups.

Supplementary Table 4 Clinicopathological characteristics statistics of HNSC patients from TCGA.
