# Supplementary figures and images for: P2RY14 Is a Potential Biomarker of Tumor Microenvironment Immunomodulation and Favorable Prognosis in Patients With Head and Neck Cancer
Source: Front Genet. 2021 Jul 8;12:670746. doi: 10.3389/fgene.2021.670746 (PMC8297391; doi:10.3389/fgene.2021.670746)

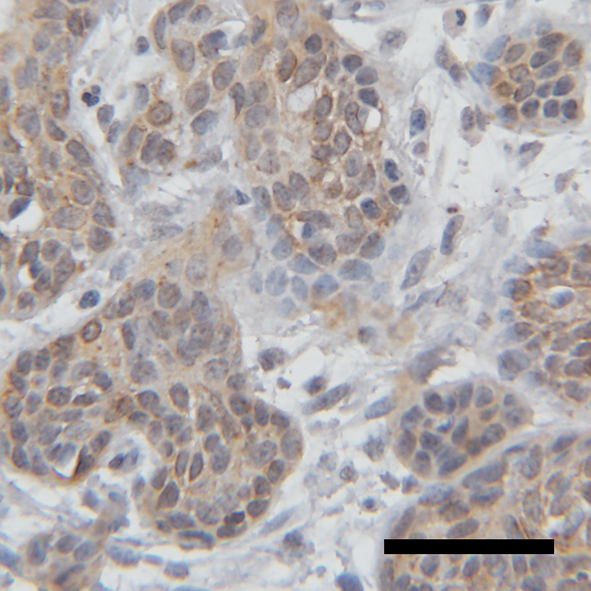

Supplement: Supplementary Figure 1 — Representative immunohistochemical staining images of P2RY14 in tumor cells from HNSC patients (scale bar: 50 μm). [file Image_1.TIF]

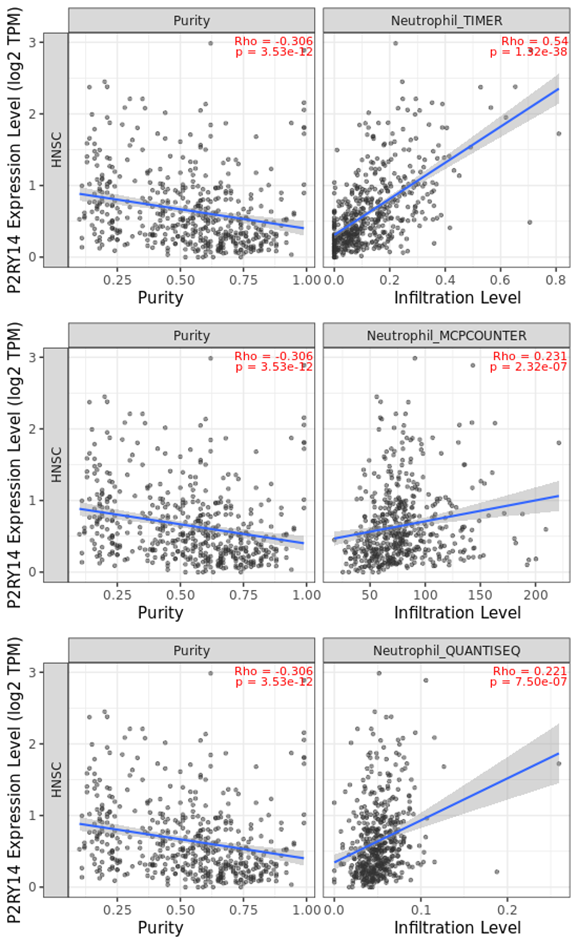

Supplement: Supplementary Figure 2 — Scatter plots showing the correlation of P2RY14 and neutrophils in head and neck cancer in the database using TIMER, MCPCOUNTER, and QUANTISEQ methods. [file Image_2.TIF]
